# Shear-mediated contributions to the effective properties of soft acoustic metamaterials including negative index

**DOI:** 10.1038/srep18562

**Published:** 2015-12-21

**Authors:** Derek Michael Forrester, Valerie J. Pinfield

**Affiliations:** 1 Loughborough University, Chemical Engineering, Loughborough, LE11 3TU, United Kingdom

## Abstract

Here we show that, for sub-wavelength particles in a fluid, viscous losses due to shear waves and their influence on neighbouring particles significantly modify the effective acoustic properties, and thereby the conditions at which negative acoustic refraction occurs. Building upon earlier single particle scattering work, we adopt a multiple scattering approach to derive the effective properties (density, bulk modulus, wavenumber). We show,through theoretical prediction, the implications for the design of “soft” (ultrasonic) metamaterials based on locally-resonant sub-wavelength porous rubber particles, through selection of particle size and concentration, and demonstrate tunability of the negative speed zones by modifying the viscosity of the suspending medium. For these lossy materials with *complex* effective properties, we confirm the use of phase angles to define the backward propagation condition in preference to “single-” and “double-negative” designations.

Acoustic propagation through suspensions of particles is relevant for biomedical^1^ and process monitoring applications^2^ and has more recently become significant in the context of the development of acoustic (or ultrasonic) metamaterials^3^,^4^,^5^. Whilst many models of acoustic propagation consider the fluid suspending phase to be inviscid (adopting a Mie scattering model including only acoustic waves^3^), many real fluids are in fact viscous and support shear wave propagation over short distances^6^,^7^. The shear waves are dissipative and decay in a few wavelengths, in a distance of the order of microns at MHz frequencies. However, they modify the effective acoustic properties of the suspension, both by viscous dissipation^6^,^7^, and by their influence on neighbouring particles^8^,^9^. Luppé *et al.*^10^ recently developed a modified multiple scattering model which includes these influences; its predictions for inhomogeneous materials have not previously been explored. Here, we apply it to sub-wavelength particulate suspensions, deriving analytical results for the additional shear effects on the effective properties of the material. Acoustic metamaterials enable particular acoustic properties to be achieved by design for a variety of purposes; for example acoustic absorption^11^, acoustic focussing^12^ and sub-wavelength imaging^13^/superlensing^14^, and cloaking^15^. One category of acoustic metamaterials is that of locally resonant materials^16^,^17^,^18^,^19^,^20^ which incorporate structures (e.g. particles or Helmholtz resonators) which are resonant in the frequency range of interest, but where the structures themselves are much smaller than the wavelength in the embedding matrix. Thus, the principles of homogenisation are applicable and the material can be considered to behave as an effective homogenous medium. The combined effects of the large scattering responses of many resonant particles lead to anomalous properties such as negative effective density and/or bulk modulus, with consequential impact on the effective phase speed in the material, including non-propagating and negative phase velocity conditions. For particles, monopolar resonances are associated with the effective bulk modulus, and dipolar resonances modify the effective density; these combine to produce the effective phase speed in the system. Overlapping regions of negative effective density and bulk modulus can lead to systems where the phase speed is negative^16^,^17^,^18^,^19^. Recently, “soft” acoustic metamaterials have been proposed, where the matrix is a fluid or gel, and the sub-resonant scatterers are compressible particles^3^,^4^,^5^. Determination of the effective properties of the metamaterials has been carried out by strain-tensor based homogenisation schemes^21^ or multiple scattering models^3^,^18^,^20^,^22^. However, the majority of studies considered only lossless systems with *real* effective properties, and consider only the *acoustic* scattering (Mie scattering). By extending the analysis of effective properties to *complex* values, Dubois *et al.*^23^ developed criteria for the complex effective density and bulk modulus which lead to negative phase speed, in terms of the phase angles of these properties. Although some workers have estimated complex effective properties for their metamaterials, these have not incorporated the losses due to shear waves and viscous dissipation in the surrounding fluid^3^,^4^.

In this theoretical study, we use a multiple scattering formulation, and consider lossy materials (complex wavenumbers), and complex scattering coefficients. We incorporate the effects of shear waves and viscous dissipation on the effective properties of a medium of particles suspended in a fluid, for both non-resonant solid particles and suspended sub-wavelength resonant scatterers. We demonstrate significant shear-mediated effects and establish their consequences for regimes of negative phase speed.

## Results

**Figure 1:**
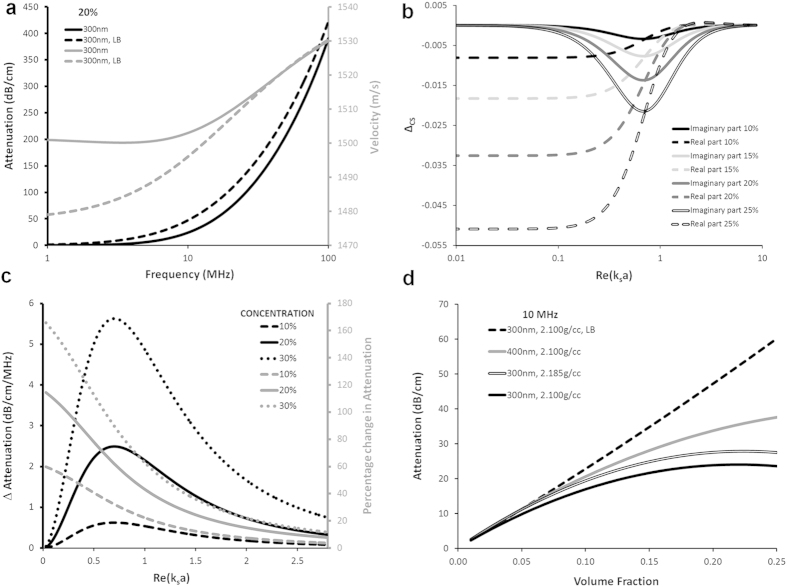
Effective properties of 300 *nm* diameter silica in water suspensions at long wavelength (non-resonant), considering the contribution of the influence of scattered shear waves on neighbouring particles. Scattering coefficients were calculated using the *ECAH* formulation which includes thermal and shear scattered waves. (**a**) Effective attenuation and speed according to the Lloyd/Berry model (dashed lines) and the modified Lloyd/Berry model (equation 1). (**b**) Additional contribution to the effective properties of multiple scattering of shear waves, 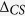, as a function of 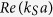 at various concentrations. 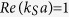 corresponds to a frequency of 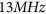. (**c**) Reduction in effective attenuation/frequency as a function of 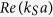 to multiple scattering of shear waves. (**d**) The effective attenuation as a function of concentration (volume fraction) showing the effect of particle size and density at 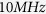 (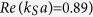.

We base our findings on a long history of developments in scattering theory; notably, the early work of Rayleigh^24^, from which single scatterer systems were described, followed much later by the successful Epstein,Carhart, Allegra, Hawley (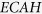) model^6^,^7^. Advancements in the field saw the Waterman/Truell^25^ (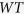) and Lloyd/Berry^26^ (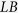) models emerge and most recently in 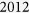 the backbone for a mathematical outlay by Luppé *et al*^10^. For silica particles in water, we examine the long wavelength condition both inside and outside the particles 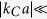, 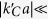 but consider the region in which shear effects are known to be significant, 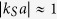. Here, 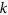 represents the wavenumber in the fluid phase, subscripts 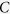 and 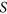 indicate compressional and shear wave modes respectively, and 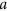 is the radius of the particles. In particular we investigate the effects of scattered shear waves on neighbouring particles, as incorporated in the second term of equation (1), 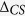, of the Methods section. Therefore a particle size (diameter) of 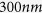 was chosen. Figure 1a shows that the effective attenuation and speed converge with that predicted by the Lloyd/Berry model at high frequency, 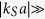, where the shear waves dissipate very close to the particle, and 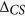 is negligible. Figure 1b further indicates that the greatest effect on attenuation (through *Im* (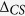)) occurs around the condition 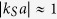, there is a constant offset on the speed at the low frequency limit (through *Re*(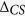)). The reduction in effective attenuation per wavelength due to the shear wave multiple scattering effects is maximal at the condition 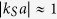 (see, Fig. 1c), and increases quadratically with concentration. The effective attenuation varies parabolically with concentration (Fig. 1d) when the additional shear effects are included and in the region 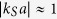, in contrast to the near-linear Lloyd/Berry predictions. An increase in radius or an increase in density increases the effective attenuation at these conditions.Although the influence of shear waves on neighbouring particles is determined by the magnitude of 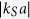, since this dimensionless parameter affects the amplitude of the shear waves produced at any one particle, it is also affected by the inter-particle separation, and therefore by the concentration. Multiple shear wave scattering effects are observed when the condition (equation (2) - see Methods section) is satisfied; the shear waves have a longer decay length than the distance between particles, and therefore can be rescattered by nearby particles. These shear wave effects must therefore be considered for relatively concentrated suspensions operating in the region 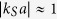 (and the long wavelength 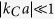, 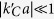 situations).

Having demonstrated the effects of shear scattering on non-resonant scatterer suspensions, we now consider acoustic metamaterials where the scatterers are resonant (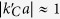), although the homogenisation condition (long wavelength outside the scatterers) is satisfied with (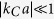). Brunet *et al.*^3^ recently demonstrated negative phase velocity conditions for a soft “suspension” of 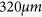 diameter porous silicone rubber particles in a polymer gel (properties like water), at 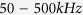. They compared their experimental data with the Rayleigh (acoustic only)/LB model. In their conditions 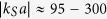 and therefore shear wave contributions are expected to be negligible, which allows good agreement to be found with the Rayleigh/LB model. The scattering model enables the prediction of negative phase velocity regions, providing a useful tool for the design of metamaterials. Here, by considering a different frequency and particle size region, where shear effects are significant, we show the importance of including them in the scattering model, and that a new class of tunable metamaterials can be constructed based on these shear effects.

**Figure 2:**
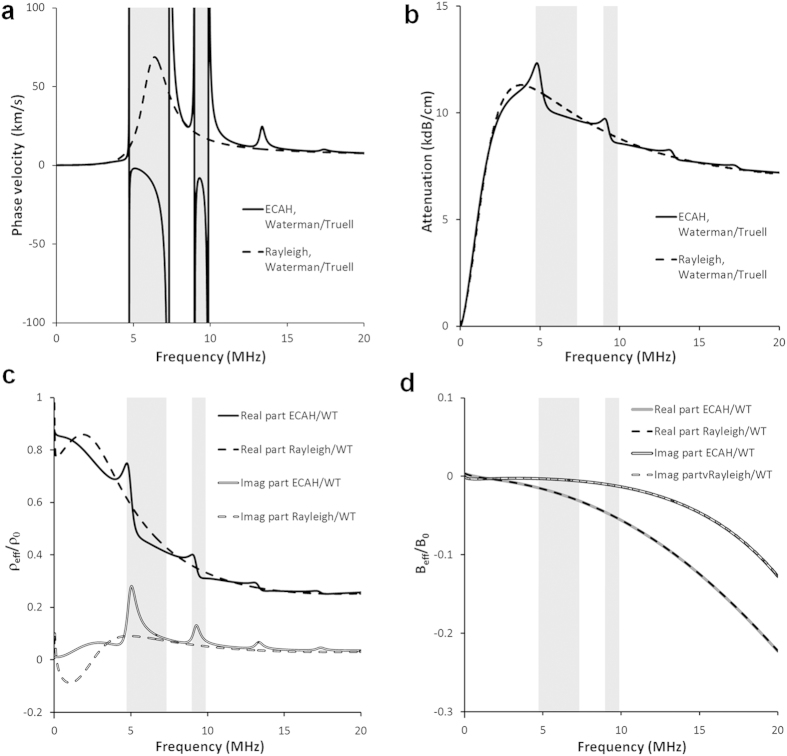
Effective properties of 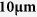 diameter porous silicone rubber in water suspensions at 30% *v/v* . Comparison is made between the scattering model predictions for effective phase velocities and attenuations without shear effects (Rayleigh, acoustic-only scattering coefficients) and with shear effects (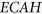 scattering coefficients, including thermal and shear effects). Both are obtained using the Waterman/Truell model. (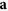) Phase velocity. (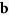) Attenuation. (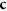) Effective density ratio. (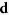) Effective bulk modulus ratio. Shading shows regions of negative phase velocity in the 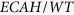 model.

In order to ensure shear effects are significant (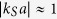) whilst also satisfying the resonance (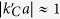) and homogenisation (long wavelength 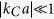) conditions, we have selected a particle diameter of 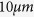 and frequency range 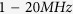 for porous rubber particles that are similar to those studied by Brunet *et al.*^3^. The speed of sound in the particles remains constant independent of pore size in the long wavelength regime that we study here. In the Supplementary Information a comparison of finite element modelling of ultrasound transmission through silicone rubber to the experimental work of Zimny *et al* 5. describing the mechanical properties and sound speeds in porous PDMS is given, demonstrating that the bulk and shear moduli remain constant, independent of pore size as long as the volume fraction of pores is maintained (Figure S1). Thus, reducing particle and pore size can still produce ultra-slow materials at the frequencies examined herein. Properties of all materials are given in Table S1 (Supplementary Information). Figure 2a shows the phase speed predicted for suspensions of these locally resonant porous silicone rubber particles in water at 30% *v/v*, using the Rayleigh model (acoustic only) and 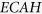 model (with shear and thermal waves). The Rayleigh model predicts no negative phase velocity bands, whereas the 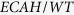 model predicts negative phase velocity in the frequency range 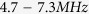 and 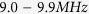 (shaded on Fig. 2). The attenuation predicted by 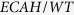 (Fig. 2b) displays peaks due to the resonance of the particles. These peaks are associated with the dipole resonance, since they coincide with regularly-spaced peaks in the effective density (Fig. 2c). The bulk modulus (relating to monopole resonance) is not significantly affected by the new shear effects (Fig. 2d). No peaks are observed in attenuation or effective density in the Rayleigh model predictions (Fig. 2b,c), implying that they arise due to shear effects. The results indicate that shear effects must be considered in the design of soft acoustic metamaterials in the region 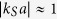. Further, we propose a new category of soft acoustic metamaterials based on the *exploitation* of shear effects, enabling new frequency regimes of negative velocity to be designed.

**Figure 3:**
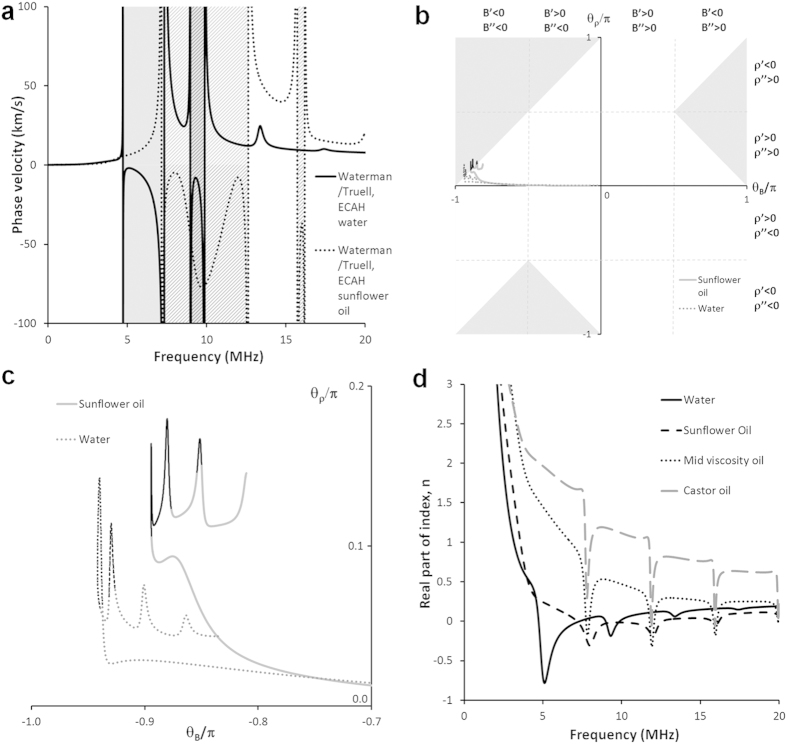
Effective properties of 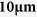 diameter porous silicone rubber 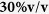 suspensions in fluids of different viscosity, using the 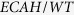 model. (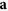) Phase velocity. Shading shows regions of negative phase velocity: grey for water, mesh for sunflower oil. (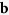) Phase angle of effective density and bulk modulus for suspensions in water (solid lines) and sunflower oil (dotted lines). (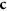) Phase angle of effective density versus phase angle of effective bulk modulus for suspensions in water (dotted lines) and sunflower oil (solid lines). The black sections in each case are where negative phase velocity occurs. Shaded regions are regions where negative velocity is expected according to the analysis of Dubois *et al.*^23^ (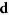) Real part of the index.

These results also imply that the acoustic properties can be manipulated by modifying the properties of the suspending phase, particularly its viscosity. To explore this effect we have investigated the same particles in four different materials: water, sunflower oil, castor oil, and a reduced-viscosity castor oil (Table S1). Figure 3a shows that the frequency bands in which negative phase velocity is observed (shaded areas) are shifted and expanded for sunflower oil compared with water suspensions, demonstrating that the increased viscosity (and possibly the reduced density) of the suspending phase has a marked impact on the negative velocity frequency band. Figure 3a shows that the frequency bands in which negative phase velocity is observed (shaded areas) are shifted and expanded for sunflower oil compared with water suspensions, demonstrating that the increased viscosity (and possibly the reduced density) of the suspending phase has a marked impact on the negative velocity frequency band. Figure S2 (Supplementary Information) shows how the negative velocity frequency bands are affected by the change in the suspending phase, and by particle size. At higher viscosity and smaller particle size, the frequency bands occur at higher frequency, with some band splitting. Figure S3 further demonstrates the influence of concentration on the backward propagation bands. As expected, there exists a minimum concentration threshold above which negative phase velocity is observed. A further increase in concentration leads in general to a reduction in the low frequency limit of the negative velocity band, and its broadening. Further discussion is in the Supplementary Information.

Following Dubois *et al.*^23^, we plot the phase angles of the *complex* effective properties (density, bulk modulus) in order to identify forward/backward propagation conditions (Fig. 3b,c). The phase angles associated with negative velocity (backward propagation) in our study coincide with those identified by Dubois *et al.*^23^ (shaded regions). For further insight, Fig. S5 (Supplementary Information) shows the phase angles plotted against frequency, and also demonstrates the shift in the dipole resonances between sunflower oil and water suspensions. The relationship of negative velocity conditions to the real/imaginary parts of the effective properties is considerably more complicated (see the labelled sub-quadrants in Fig. 3b), and therefore the use of phase angles is preferred over designations such as “negative” or “double negative” which are meaningful only for real effective properties.

## Discussion

There are many forms of metamaterials; some examples of which are those made up of composite media^27^, electronic negative index 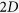 materials^28^,^29^,^30^, ferromagnets^31^, novel superconductor structures^32^,^33^,^34^,^35^,liquid foams^36^, and recently colloidal suspensions^3^,^37^. Here we have developed a new and highly promising form of the latter, with shear-wave mediated phenomena, that could supersede conventional metamaterials (that typically have narrow operational bandwidths) to cover a large ultrasonic frequency range. Thus, we identify the potential for the design of “tunable” acoustic metamaterials, by exploiting the shear effects described here. We have seen that the frequency bands for negative velocity conditions are influenced by the viscosity of the suspending phase; Fig. 3d illustrates the index for the different suspending oils. Moreover, the same effect can be achieved by a change of temperature; through which the viscosity of an oil can have strong variation.Thus it may be possible to switch on or off the negative phase velocity conditions by use of this phenomenon. We have demonstrated the influence of shear effects on the acoustic propagation in concentrated suspensions and the implications for metamaterial design. For non-resonant particles, the effects of shear waves on neighbouring particles leads to a marked reduction in effective attenuation. For locally resonant systems, we have shown that shear effects must be accounted for when designing soft acoustic metamaterials with negative phase velocity frequency bands. Those bands are strongly affected by the viscosity and properties of the suspending phase. The phase angle of the complex effective density and bulk modulus is a reliable indicator of the existence of negative phase velocity behaviour and is used in preference to the designations “negative” and “double negative” materials in these lossy systems. We propose the design of tunable metamaterials using temperature-dependent viscosity to modify the negative phase velocity bands. The results suggest the potential for constructing colloidal super-lattices of soft metamaterials with different suspending phases (see Figure S4), exploiting the overlapping backward propagation bands to design novel acoustic behaviour.

## Methods

The multiple scattering model of Lloyd and Berry^26^ (denoted 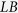) is used to obtain the effective acoustic properties of a suspension of particles, with monopole and dipole partial wave orders, and up to second order in concentration, as stated by Challis *et al.*^38^. This is the first term in equation (1). An additional term (the second term in equation (1)) is included at the second order in concentration, following the expression obtained by Luppé *et al.*^10^ Their model incorporates the effects of scattered thermal and shear waves being rescattered at neighbouring particles, and reconverted into the acoustic mode. Here we present predictions of the effective acoustic properties (attenuation, speed) from their model for the first time. We therefore write,


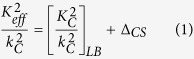


where 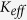 denotes the effective wavenumber in the whole medium. The multiple scattering model is based on the single particle scattering coefficients (see Supplementary information); here, the formulations of Rayleigh^24^, and Epstein-Carhart/Allegra-Hawley (ECAH)^6^,^7^ are used. We refer to the “Rayleigh” formulation for scattering with only acoustic wave modes (no thermal or viscous losses), whereas the ECAH scattering models incorporate losses caused by thermal and shear waves produced at the particle which decay in the region around it. From the work of Luppé *et al.*^10^, we have derived an analytical expression for the additional term 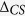 arising from the compressional-shear-compressional mode conversions, for the case of solid particles suspended in a fluid. In that case, the shear wave scattering in the dipole partial wave order is the significant contribution. The details of this new term are given in the Supplementary Information, along with the associated scattering coefficients for the dipole partial wave with incident compressional or shear wave. For the metamaterial calculations, the Waterman/Truell (WT)^25^ multiple scattering model was used because it can be separated into effective density and bulk modulus components)^3^,^39^ (see Supplementary Information).

The conditions for metamaterial behaviour, and for the significance of viscous effects, can be established using the dimensionless parameters relating particle size to the wavelength of each wave mode. Homogenisation of the metamaterial (in which it behaves as an effective homogeneous medium) is valid when the wavelength outside the particles is much larger than the particle. For spherical particles of radius 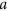, this condition is 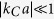. When the particles are resonant, the wavelength inside the particle is of the same order as the particle size, thus 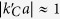. Significant viscous losses occur when the shear wavelength outside the particle is of the same order as the particle size^2^ 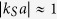. In addition, scattered shear waves influence neighbouring particles (through hydrodynamic effects) when the shear decay distance is longer than the interparticle separation^9^, thus


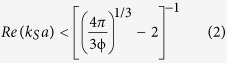


These criteria can be used to identify the regions in which viscous losses become significant, shear mode conversion becomes important, or to design metamaterials which exploit shear effects. In the main text we presented examples of each of these regions. Calculations were carried out in Matlab to derive the effective properties of suspensions of silica particles (a concentrated non-resonant suspension), and silicone rubber particles (near resonance), to explore the contribution of shear effects on those properties. The physical properties of the constituent materials are given in Supplementary Information, Table S1.

## Additional Information

**Data Availability**: The data (held in the Loughborough University repository with DOI:10.17028/ rd.lboro.2003814) reported here can be obtained by contacting the authors.

**How to cite this article**: Forrester, M. and Pinfield, V. Shear-mediated contributions to the effective properties of soft acoustic metamaterials including negative index. *Sci. Rep.* **5**, 18562; doi: 10.1038/srep18562 (2015).

## Supplementary Material

Supplementary Information
