# Erratum: Shear-mediated contributions to the effective properties of soft acoustic metamaterials including negative index

**DOI:** 10.1038/srep34120

**Published:** 2016-09-30

**Authors:** Derek Michael Forrester, Valerie J. Pinfield

Scientific Reports 5: Article number: 1856210.1038/srep18562; published online: 12 21 2015; updated: 09 30 2016

In this Article, the values of two inequalities have been omitted in the Results section during typesetting.

‘‘For silica particles in water, we examine the long wavelength condition both inside and outside the particles | *k*_*C*_*a* | ≪ 1, | *k*′_*C*_*a* | ≪ but consider the region in which shear effects are known to be significant, | *k*_*S*_*a* | ≈ 1.’’

should read:

‘‘For silica particles in water, we examine the long wavelength condition both inside and outside the particles | *k*_*C*_*a* | ≪ 1, | *k*′_*C*_*a* | ≪ 1 but consider the region in which shear effects are known to be significant, | *k*_*S*_*a* | ≈ 1.’’

In addition,

‘‘Figure 1a shows that the effective attenuation and speed converge with that predicted by the Lloyd/Berry model at high frequency, | *k*_*S*_*a* | ≫. where the shear waves dissipate very close to the particle, and is negligible.’’

should read:

‘‘Figure 1a shows that the effective attenuation and speed converge with that predicted by the Lloyd/Berry model at high frequency, | *k*_*S*_*a* | ≫ 1, where the shear waves dissipate very close to the particle, and is negligible.’’

