# Supplementary material for: Shear-mediated contributions to the effective properties of soft acoustic metamaterials including negative index
Source: Sci Rep. 2015 Dec 21;5:18562. doi: 10.1038/srep18562 (PMC4685261; doi:10.1038/srep18562)
Supplement: Supplementary Information [file srep18562-s1.pdf]

Shear-mediated contributions to the effective properties of soft acoustic metamaterials including negative index

Supplementary Information

D. M. Forrester and V. J. Pinfield

Chemical Engineering Department, Loughborough University, Loughborough, Leicestershire, UK, LE11 3TU

Theory

The Lloyd/Berry multiple scattering model[1] (denoted LB), to second order in concentration, and including only monopolar and dipolar partial wave orders (zero and one) is given by

$$\left[\frac{K_C^2}{k_C^2}\right]_{LB} = 1 - \frac{3i\phi}{(k_C a)^3}\left[T_0^{CC} + 3T_1^{CC}\right] - \frac{27\phi^2}{(k_C a)^6}\left[T_0^{CC}T_1^{CC} + 2T_1^{CC}T_1^{CC}\right] \qquad \text{(S1)}$$

where ϕ is the volume fraction of scatterers, a is the radius of the (spherical) scatterer, and T_n^{ij} are the single particle scattering coefficients (transition factors). These are for partial wave order n for incident wave of mode $i = C, T \text{ or } S$ and scattered wave of mode $j = C, T \text{ or } S$, denoting compressional (acoustic), thermal and shear wave modes respectively. The Waterman/Truell (WT) multiple scattering model (used for metamaterial calculations) omits the final term in $T_1^{CC}T_1^{CC}$ in the second order in concentration.

The transition factors can be derived by solving the boundary equations at the scatterer surface for each partial wave order. In the case where only acoustic scattering is considered (the Mie scattering or Rayleigh scattering form), the scattering coefficients can be obtained from the Rayleigh formulation[2], where thermal and shear waves are omitted. In this work, the contributions of shear-mediated effects have been investigated and therefore we have also calculated the effective properties using the transition factors obtained from the Epstein/Carhart[3], Allegra/Hawley[4] (denoted

ECAH) formulation in which thermal and shear scattered wave modes are included both inside and outside the scatterer. The conversion of energy from the compressional wave mode into thermal and shear waves leads to dissipation by thermal and viscous mechanisms around the particle. Further details and applications of the Lloyd/Berry- Epstein/Carhart, Allegra/Hawley model can be found in a review paper by Challis et al.[5]

The shear waves produced by scattering of the incident compressional wave mode decay in the region around the scatterer. However, at lower frequency, the decay length is longer since the wavenumber for shear waves in a fluid is given by

$$k_S = (1+i)\sqrt{\frac{\omega\rho}{2\eta}} \qquad \text{(S2)}$$

Also at higher particle number density, the distance between particles is smaller. Hence under some conditions (low frequency, high concentration), the shear waves reach neighbouring particles and are rescattered, thereby producing waves of each of the three modes[6-7]. The scattered compressional waves contribute to the coherent compressional wave field, and therefore modify the effective compressional (acoustic) wavenumber in the material. This is the cause of the additional term derived in a new multiple scattering formulation by Luppé et al[8]. We have identified that the principal contribution to these additional shear effects arises from the dipole partial wave order, with transition factors for compressional-shear mode conversion and vice versa. We have derived the analytical form for the dominant contribution to the effective wavenumber from the formulation of Luppé et al.[8]

$$\Delta_{CS} = -\frac{27\phi^2}{(k_C a)^6}\frac{k_C^3(ik_S b)}{k_S(k_C^2 - k_S^2)}T_1^{CS}T_1^{SC}X \qquad \text{(S3)}$$

$$X = k_C b j_0'(k_C b)h_0(k_S b) - k_S b j_0(k_C b)h_0'(k_S b)$$

Note that there is an additional term in *X* due to second partial wave order Bessel and Hankel functions, but that term is negligible compared with those stated here.

This additional multiple scattering contribution due to shear wave modes requires the transition factors T_1^{CS} and T_1^{SC} as previously noted. The former, for incident compressional wave, can be obtained directly from the Epstein/Carhart, Allegra/Hawley formulation. The latter represents the "amplitude" of the scattered compressional wave due to an incident shear wave; the incident shear wave takes a similar form to the scattered shear wave in the ECAH formulation. The transition factor can be obtained by solution of the boundary equation matrix in the same way as in the ECAH method, replacing the incident wave terms.

We have derived analytical expressions for the transition factors in the case of non-resonant scatterers where the wavelength of the compressional wave is much larger than the size of the scatterer both inside and outside the particle. These are the leading terms in a series expansion in $k_C a$, whilst assuming that $k'_C a$ is of the same order as $k_C a$ (the prime indicates inside the particle). Thus,

$$T_1^{CS} = -\frac{k_C a(\hat{\rho}-1)}{k_S a \cdot \left(3h_2(k_S a) - 2(\hat{\rho}-1)h_0(k_S a)\right)} \quad \text{(S4)}$$

$$T_1^{SC} = -\frac{2i}{3}(k_C a)^2 k_S a(\hat{\rho}-1)\frac{\left[h_2(k_S a)\, j_0(k_S a) - h_0(k_S a)\, j_2(k_S a)\right]}{3h_2(k_S a) - 2(\hat{\rho}-1)h_0(k_S a)} \quad \text{(S5)}$$

For the metamaterial calculations the wavelength is long outside the scatterer, $|k_C a| << 1$ but the particles are resonant, $|k'_C a| \sim 1$. The acoustic properties of the metamaterial can be defined by an effective density and bulk modulus, which are related to the effective wavenumber by

$$\frac{K_{eff}^2}{k_c^2} = \left(\frac{\rho_{eff}}{\rho_0}\right)\left(\frac{B_0}{B_{eff}}\right) \quad \text{(S6)}$$

where ρ_0 and B_0 denote the density and complex bulk modulus of the continuous phase respectively, and the subscript *eff* denotes the effective properties of the medium. Since the wavenumber in the continuous phase is complex (lossy material), the density can be taken as the "true" density of the

material, but the complex bulk modulus must be defined through $k_c^2 = \omega^2 \left(\rho_0 / B_0 \right)$. This is equivalent to assigning all losses to the bulk modulus. The effective density and bulk modulus have been calculated following the procedure of Parnell and Abrahams[9], using the Waterman/Truell multiple scattering formulation. Thus,

$$\frac{\rho_{eff}}{\rho_0} = 1 - \frac{3i\phi}{(k_C a)^3}\left(3T_1^{CC}\right) \tag{S7}$$

$$\frac{B_{eff}}{B_0} = \left\{1 - \frac{3i\phi}{(k_C a)^3}\left(T_0^{CC}\right)\right\}^{-1} \tag{S8}$$

The index has been defined as

$$n = \frac{K_{eff}}{k_C} \tag{S9}$$

and is complex since both wavenumbers are complex.

Results

Material Properties

The properties of the materials used in the simulations are presented in table S1. Silica properties were taken from Ref 5 but with a slightly lower density, following experimental measurements. Silicone rubber properties (density, compressional wave speed and attenuation, shear modulus, from the shear wave speed) were taken from Brunet et al.[10] who measured these values; the other properties for silicone rubber were taken from Ref 11, without accounting for the porosity of the particles. The shear wave propagation in the rubber was assumed here to be lossless. Sunflower oil properties were from Ref 12. Data for castor oil was obtained from Kaye and Laby[13] (Tables 2.2.1 Densities, 2.2.3 Viscosities, 2.4.1 Speed and attenuation of sound), with the exception of the thermal

properties (heat capacity, conductivity, expansivity) which were obtained from the Engineering Toolbox.[14]

	Silica[5]	Water[5]	Porous silicone rubber[10,11]	Sunflower oil[12]	Castor oil[13-14]
c m s^{-1}	5968	1497	80	1469.9	1490
ρ kg m^{-3}	2100	997	600	920.6	950
μ N m^{-2}	3.09×10^{10}		9.6×10^{5}		
η Pa s		8.91×10^{-4}		0.054	0.7
κ W m^{-1} K^{-1}	1.6	0.595	0.2	0.17	0.18
C_P J kg^{-1} K^{-1}	729	4179	1100	1980	1800
α Np m^{-1} MHz^{-p}	2.6×10^{-10}	0.023	60000	1.15	5.3
Exponent p	2	2	1.5	1.77	2
β_T K^{-1}	1.35×10^{-6}	2.1×10^{-4}	2.5×10^{-4}	7.1×10^{-4}	7.0×10^{-4}

Table S1. *Material properties used in the simulations.*

The size of the pores in the silicone rubber were found to have little influence on the speed of sound in the long wavelength limits discussed here. Finite element modelling was conducted using the transient pressure acoustics module of Comsol Multiphysics (version 5.1) to determine these speeds. Zimny et al. experimentally found the speeds of sound in soft porous silicone rubber, with a value of $80m/s$ when the porosity was $\Phi \approx 20-40\%$ [16]. They demonstrated that the speed of sound in the porous rubber can be described as a function of the porosity: $c = c_0 / \sqrt{(1-\Phi)(1+3K\Phi/4\mu)}$, with $c_0 = 1000m/s$ in non-porous PDMS. Using this equation we plot the speed of sound as a function of porosity, solid line in Figure S1, and also the results from the finite element (FE) modelling (black circles). For simplicity the pores are taken as spheres, with a square lattice arrangement, in the modelling. As a result, at low pore density the FE model overestimates the speed of sound as the channels between the pores are quite large and linear. As the porosity increases the channels become squeezed and a better comparison to the results of Zimny emerges. We are interested in structures with a porosity that results in the lowest possible speeds and show in Figure S1 that for pore radii of $1.25, 2.5, \& 5\mu m$ and pore volume fraction of 0.35 that c remains constant (overlap in Figure S1), indicating that the shear and bulk moduli remain the same independent of pore size. Thus, in the

frequency regimes that we examine for the acoustic metamaterials, scaling the pore size from a $10\mu m$ diameter downwards in a slab of PDMS has no influence on the speed of sound as long as the volume fraction of pores is adjusted accordingly.

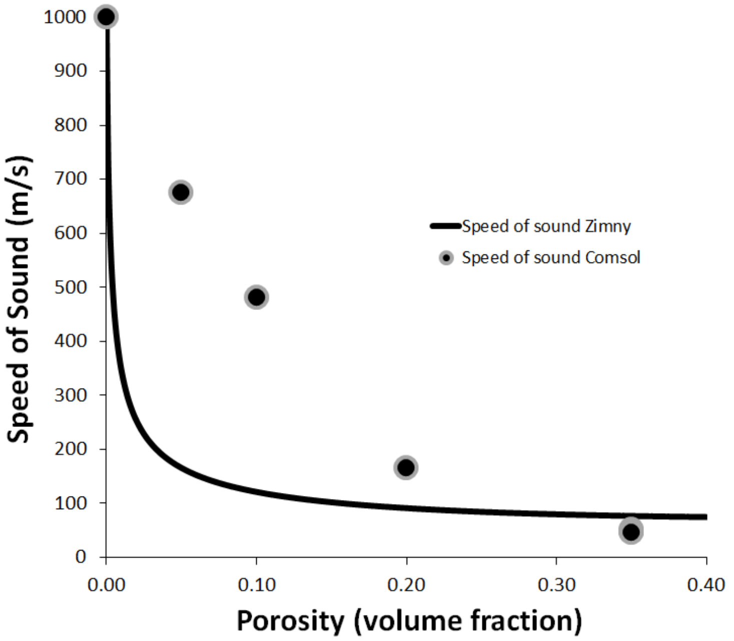

Figure S1. *The speed of sound is shown as a function of porosity (thick line: Zimny relation*[16]*, circles: Comsol FEM). The Comsol model simulates an ultrasonic emitter and receiver with a* $4mm$ *sample of PDMS in-between (all in an air-environment) such that for a non-porous sample it takes* $4\mu s$ *for a* $0.5MHz$ *plane pressure wave to propagate from the emitter to the receiver. As the porosity increases there is a large decrease in sound speed. The FEM is conducted for pore sizes of* $5\mu m$ *radius, except at* 35% *where pore radii of* $1.25, 2.5, \& 5\mu m$ *are examined (overlapping velocities in the above).*

We have discussed $10\mu m$ diameter porous silicone rubber in the context of soft acoustic metamaterials. There are a number of cases in the literature where porous PDMS has been produced at this scale. For example, Ou, Ren, and Pawliszyn fabricated $10\mu m$ thick PDMS membranes with macropores ranging from $1-2\mu m$ for use in microfluidics[17]. Currently work is underway to adapt the methods of Gorkmen[18], Zimny[16] and co-workers to create $5-10\mu m$ diameter porous silicone rubber using an adapted micro-emulsification process[19].

Negative phase velocities

Figure S2 shows the frequency ranges over which negative velocity is expected to occur for 30%v/v porous silicone rubber in various suspending fluids. The higher viscosity oils have bands at higher frequency and these become very narrow ranges. The dipole resonances are modified as the oil viscosity increases, seen by the sharpening and strengthening of the resonance structures in the effective density (Fig. S2b). Particle size also influences the negative velocity bands (Fig. S2c-d); a larger particle size reduces the frequency at which negative velocity is predicted. Figure S3 demonstrates that the emergence of negative phase velocity is a concentration-dependent phenomenon. For each particle size there is a minimum threshold concentration below which negative velocities are not predicted; this lies between 15-20%v/v for these systems. Above this threshold, an increase in concentration leads to a decrease in the lower frequency limit of the negative velocity band and a broadening of the band. An increase in particle size has a similar effect, consistent with the effects observed in Fig. S2c-d. Figure S3a-b illustrates the accompanying changes in the velocity/frequency resonance profiles, showing how the negative velocity conditions are modified as the resonance changes with particle size and concentration. The results demonstrate the potential manipulation of metamaterial properties by concentration, by particle size, and by suspending phase viscosity, due to the shear-mediated effects.

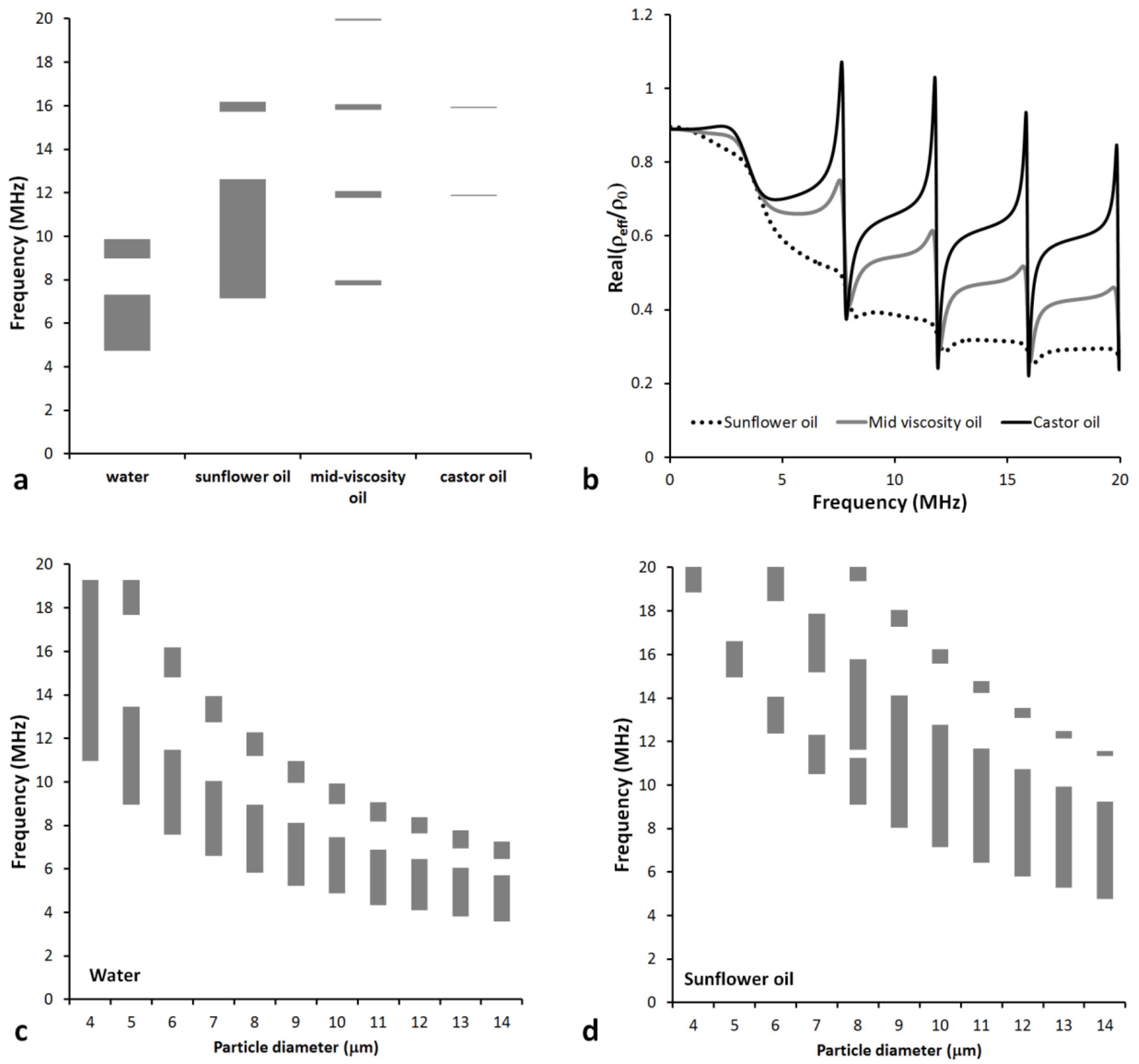

Figure S2. *The frequency dependencies of the silicone rubber suspensions.* ***a****, Frequency bands where negative phase velocity is observed for 30%v/v suspensions of 10µm diameter porous silicone rubber in different suspending phases.* ***b****, Effective density ratios for 30%v/v suspensions of 10µm diameter porous silicone rubber in various oils.* ***c****, The frequencies at which negative phase velocities occur with different particle sizes of silicone rubber in water at a volume fraction of 30%v/v.* ***d****, The same as in* ***c****, except using sunflower oil as the suspending phase.*

Since the frequency bands for negative velocity are different in different suspending phases and for different particle sizes, a colloidal superlattice is proposed (Fig. S4) in order to obtain further novel properties. Such a structure could permit broader frequency ranges for backward propagation

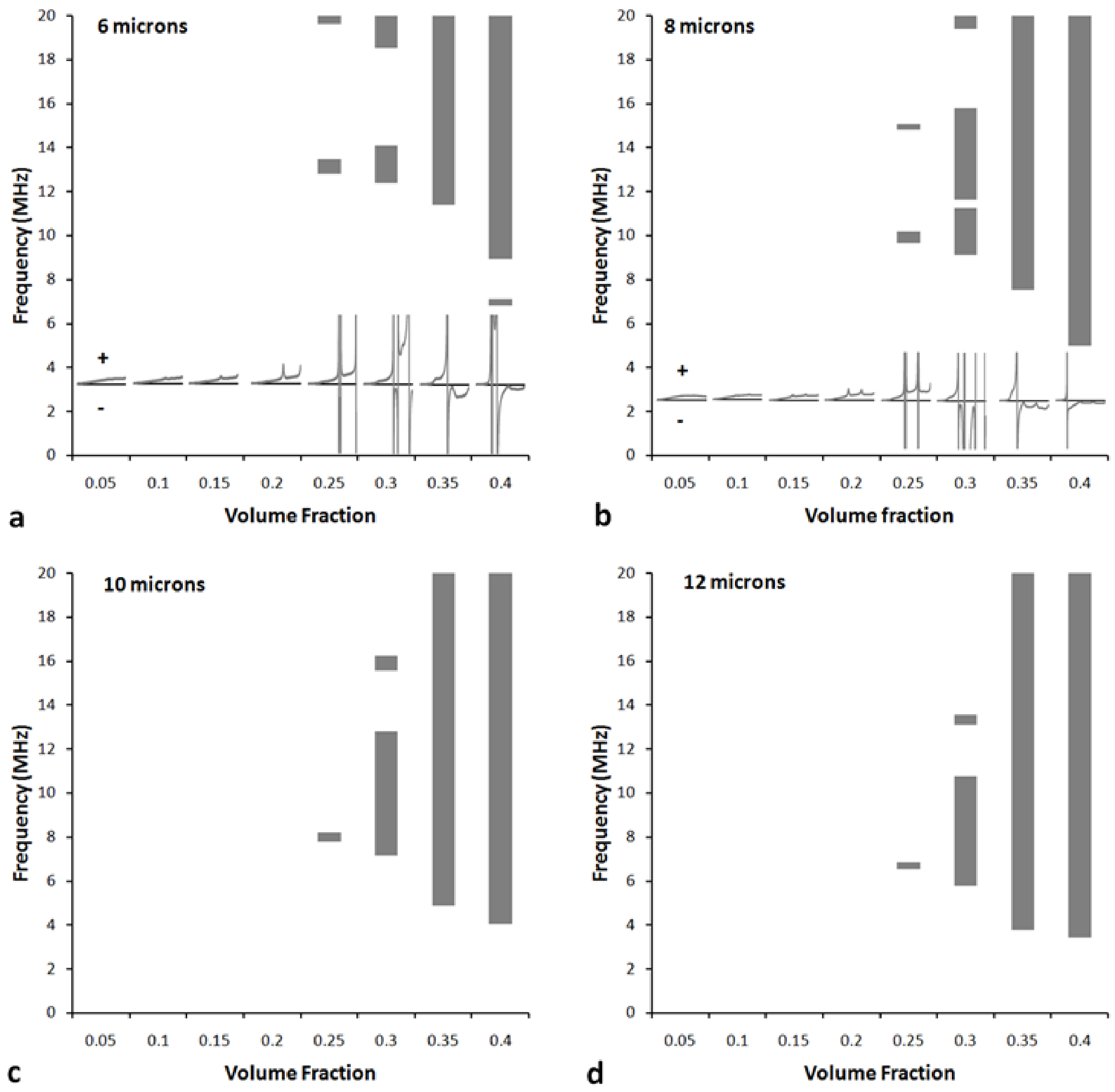

Figure S3. *The frequency bands corresponding to negative phase velocities as a function of volume fraction of silicone rubber particles in sunflower oil.* ***a***, *6 micron diameter.* ***b***, *8 micron diameter.* ***c***, *10 micron diameter.* ***d***, *12 micron diameter. Underneath* ***a*** *and* ***b*** *are the phase velocities as a function of frequency as they evolve for each volume fraction.*

conditions, or could exploit the resonances of the layered structure itself to permit only narrow frequency bands of transmission or reflection. Viscosity changes controlled by temperature enable a simple method for tuning the refractive index in the superlattice. In its raw state it is a tunable, viscoelastic, colloidal metamaterial. These metamaterials of interfacially separated oil/water structures merit further investigation.

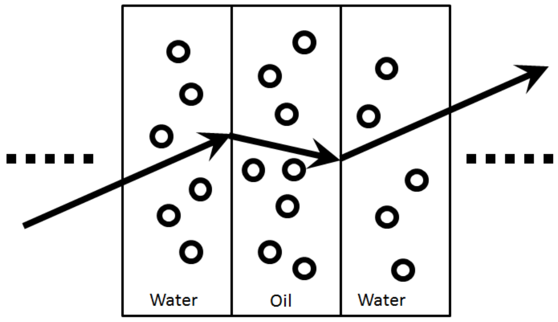

Figure S4. *Colloidal superlattice of metamaterial layers; each layer being a suspension of particles in a different suspending media.*

The phase angle of effective density and bulk modulus are used to characterise acoustic propagation characteristics in lossy systems, following Dubois et al.[15] as explained in the main text. Figure 3b-c showed how the phase angle of effective density varied with that of the effective bulk modulus in our systems. Here (Fig. S5), we plot the phase angle as a function of frequency in order to show how

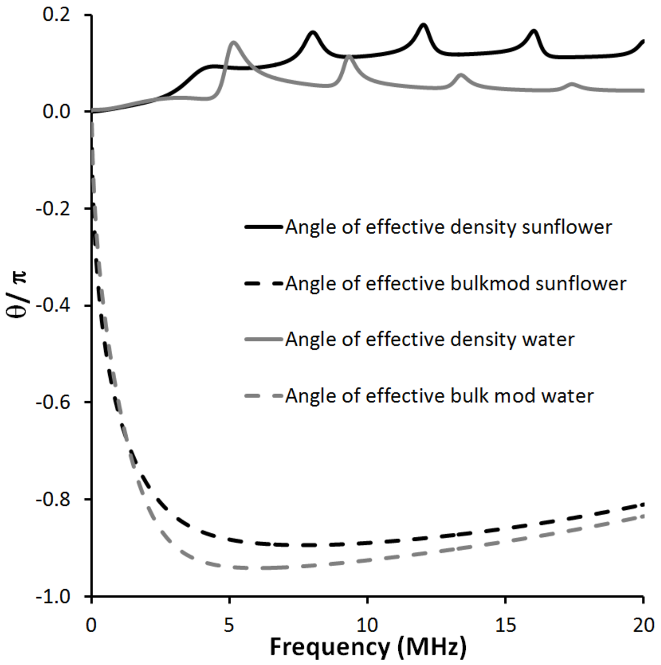

Figure S5. *Phase angle of effective density (solid lines) and bulk modulus (dashed lines) for suspensions of 10µm diameter porous silicone rubber in water (grey) and sunflower oil (black) at 30%v/v.*

the conditions under which the various propagation characteristics occurred. The effective density shows the dipole resonance conditions, with a clear shift in the frequency of the resonances as the suspending phase is changed from water to sunflower oil. The negative velocity conditions occur when the phase angles satisfy the condition[15]

$$\sin(\theta_\rho - \theta_B) < 0 . \qquad \text{(S10)}$$

References

1. Lloyd, P. & Berry, M. V. Wave propagation through an assembly of spheres IV Relations between different multiple scattering theories. *Proceedings of the physical society*, London **91**, 678-688 (1967).
2. Strutt (Baron Rayleigh), J. W. S. in The Theory of Sound (Dover Publications, New York, 1945).
3. Epstein, P. S. & Carhart, R. R. The absorption of sound in suspensions and emulsions. I. Water fog in air. *Journal of the Acoustical Society of America* **25**, 553-565 (1953).
4. Allegra, J. R. & Hawley, S. A. Attenuation of sound in suspensions and emulsions: theory and experiments. *Journal of the Acoustical Society of America* **51**, 1545-1564 (1972).
5. Challis, R. E., Povey, M. J. W., Mather, M. L. & Holmes, A. K. Ultrasound techniques for characterizing colloidal dispersions. *Reports on Progress in Physics* **68**, 1541-1637 (2005).
6. Challis, R. E. & Pinfield, V. J. Ultrasonic wave propagation in concentrated slurries – The modelling problem. *Ultrasonics* **54**, 1737-1744 (2014).
7. Hipp, A. K., Storti, G. & Morbidelli, M. Acoustic characterization of concentrated suspensions and emulsions 1. Model analysis. *Langmuir* **18**, 391-404; 391 (2002).
8. Luppé, F., Conoir, J. M. & Norris, A. N. Effective wave numbers for thermo-viscoelastic media containing random configurations of spherical scatterers. *Journal of the Acoustical Society of America* **131**, 1113-1120 (2012).

9. Parnell, W. J. & Abrahams, I. D. Multiple point scattering to determine the effective wavenumber and effective material properties of an inhomogeneous slab. *Waves in Random and Complex Media* **20**, 678-701 (2010).

10. Brunet, T. *et al*. Soft 3D acoustic metamaterial with negative index. *Nature Materials* **14**, 384-388 (2015).

11. AZO materials. http://www.azom.com/properties.aspx?ArticleID=920 Accessed on 24/6/2015.

12. McClements, D. J. & Povey, M. J. W. Scattering of ultrasound by emulsions. *Journal of Physics D: Applied Physics* **22**, 38-47; 38 (1989).

13. Kaye and Laby Tables of Physical & Chemical Constants (16th edition 1995). Kaye & Laby Online. Version 1.0 (2005) www.kayelaby.npl.co.uk

14. Engineering toolbox. http://www.engineeringtoolbox.com/ Accessed on 24/6/2015.

15. Dubois, J., Aristegui, C. & Poncelet, O. Spaces of electromagnetic and mechanical constitutive parameters for dissipative media with either positive or negative index. *J. Appl. Phys*. **115**, 024902 (2014).

16. Zimny, K. *et al*. Soft porous rubbers as key elements for the realization of acoustic metamaterials. *Langmuir* **31**, 3215-3221 (2015).

17. Ou, J., Ren, C. L., & Pawliszyn, J. A simple method for preparation of macroporous polydimethylsiloxane membrane for microfluidic chip-based isoelectric focusing applications. *Analytica Chimica Acta* **662**, 200–205 (2010).

18. Gokmen, M. T. *et al*. Fabrication of Porous "Clickable" Polymer Beads and Rods through Generation of High Internal Phase Emulsion (HIPE) Droplets in a Simple Microfluidic Device. *Macromolecules* **42** (23), 9289–9294 (2009).

19. Vladisavljević, G. T. & Schubert, H. Influence of process parameters on droplet size distribution in SPG membrane emulsification and stability of prepared emulsion droplets. *Journal of Membrane Science* **225**, 1–2, 15–23 (2003).
